# The Power in Rural Place Stigma

**DOI:** 10.1007/s11673-023-10260-9

**Published:** 2023-05-09

**Authors:** Christina A. R. Malatzky, Danielle L. Couch

**Affiliations:** 1 Centre for Justice, School of Public Health and Social Work, Queensland University of Technology, 149 Victoria Park Road, Kelvin Grove, Queensland 4059 Australia; 2 Monash Rural Health, Monash University, PO Box 666, Bendigo, Victoria 3552 Australia

**Keywords:** Power, Stigma, Place, Rural/rurality, Spatial/territorial stigma, Structural stigma, Health inequality, Spatial injustice, Public health, Rural health

## Abstract

The phenomenon and implications of stigma have been recognized across many contexts and in relation to many discrete issues or conditions. The notion of spatial stigma has been developed within stigma literature, although the importance and relevance of spatial stigma for rural places and rural people have been largely neglected. This is the case even within fields of inquiry like public and rural health, which are expansively tasked with addressing the socio-structural drivers of health inequalities. In this paper, we argue that developing a better understanding of rural place stigma is critical for addressing contemporary patterns of spatial injustice and health inequalities affecting rural communities globally. Drawing on international literature and examples from the reported experiences of rurally living Australians and news and other media, we present an analysis highlighting the power in rural place stigma. In doing so, we build a case for the relevance and importance of interrogating rural place stigma, especially in the fields of public and rural health, for changing the conditions within—and the broader positioning of—the rural in the public and political landscapes.

## Introduction

The symbolic meanings attached to place have tangible consequences for experiences of “living in-place” (Malatzky, Cosgrave, and Gillespie 2020a, 2). How rural places are represented and perceived has social, economic, and health consequences for people who live rurally (Eriksson 2008). Social institutions, including the media, play a key role in what Malatzky and Smith (2022) describe as the repeated devaluing and othering of the rural to “maintain the ‘centre’ of focus on the urban” by (re)creating and disseminating stigmatizing constructions of rural places and rural people. There is extensive research that articulates the impacts of stigma on health issues such as mental illness (see Livingston and Boyd 2010), HIV (see Lee, Kochman, and Sikkema 2002), and social groups—for example, people with diverse genders and sexualities (see Puckett and Levitt 2015). Some literature also details the phenomenon of spatial stigma (Halliday et al. 2020; Keene and Padilla 2014; Tyler and Slater 2018; Wacquant, Slater, and Pereira 2014). However, scant attention has been given to the impacts of stigma on rural places and those living rurally. For example, the construction of rural places as boring and rural people as backward has implications for how rural practice is perceived by health and education providers and, in turn, affects rural people’s access to key health protecting and promoting resources. Instead, analyses of spatial stigma almost exclusively focus on urban place contexts. We argue that developing a better understanding of rural place stigma is imperative for addressing contemporary patterns of spatial injustice and health inequalities that are persistent, even in some of the world’s wealthiest countries (Malatzky and Smith 2022).

What is of most interest to us in this paper is how the stigmatization of rural places (re)produces and maintains existing power relations within many contemporary societies that privilege the position of metro-orientated perspectives and interests in public debates and political decision-making (Fors 2018). These are frequently assumed to represent and work for all place contexts but disadvantage people living in rural places. In presenting our analysis of the power in rural place stigma, we take Corrigan’s (2005, 551) argument that stigma is created by socio-political forces and involves “policies of private and government institutions that restrict the opportunities of the groups that are stigmatized.” We begin by broadly conceptualizing stigma and describing the relationship between stigma and power. This is followed by an examination of spatial stigma. We highlight the almost exclusive focus on urban place contexts in the current literature and the relevance of spatial stigma to understanding the positioning of rural places and people in contemporary political struggles. We then outline a case for exploring the stigmatization of rural places. We draw on examples of rural living Australians’ published experiences, news and other forms of media, and international literature to highlight how rural people experience and reflect on rural stigma. Finally, we consider rural, community-level responses to rural place stigma and the limitations of these in the context of broader power relations. Our analysis builds a strong case for further examinations and interrogations of rural place stigma within public and rural health research and praxis.

## Stigma and Power

In his now seminal work on the subject, Erving Goffman (1963, 3, 5) defined stigma as “an attribute that is deeply discrediting . . . an undesired differentness” that affects social situations. In this context, the term comes from the Greek word *stizein*, meaning “to tattoo,” and then the Latin word *stigmat*, meaning “mark, brand” (Merriam-Webster 2020). It was originally used to describe cuts or burn marks on the skin that identified those marked (usually criminals, slaves, and traitors) as immoral or tainted people who needed to be avoided (Bos et al. 2013). Goffman (1963) describes three broadly different types of stigma: abominations of the body (such as physical deformities), individual character flaws (such as unnatural passions, weak will, and dishonesty), and tribal stigma (such as stigma associated with race, religion, or nation). A wide range of other inadequacies, in addition to the original source of imperfection/stigma, are often ascribed to individuals or groups of people who experience stigmatization.

Internalized stigma, also referred to as *self* or *felt* stigma, exists at the individual (i.e., micro) level and, in the context of mental illness, can be described as a process whereby affected individuals endorse stereotypes about mental illness, anticipate social rejection, consider stereotypes to be self-relevant, and believe they are devalued members of society (Corrigan et al. 2005; Corrigan and Watson 2002; Corrigan, Watson, and Barr 2006; Boyd Ritsher and Phelan 2004). Further distinctions have been made between *felt* and *self* stigma (Herek 2007; Herek, Gillis, and Cogan 2009). Whereas *felt* stigma describes negative consequences resulting from an individual’s awareness of how society perceives, and will likely act toward, the group to which they belong (e.g., those whose sexual orientations are other than heterosexual or those with a form of mental illness), *self* stigma refers to the process of an individual accepting society’s negative evaluation and incorporating it into their own personal value system and sense of self. Similarly, distinctions have been made between *perceived* stigma (awareness of stereotypes) and *self* stigma, with the latter being defined as: “when the person internalizes the stigma and applies it to people with mental illness in general (stereotype agreement) or to him or herself (self-concurrence)” (Corrigan et al. 2006, 882). The processes and factors involved in internalized stigma for people with mental illness have been elucidated in several models, including Corrigan and Watson’s (2002) situational model and Link et al.’s (1989) modified labelling theory.

Examining the relevance to and effects of stigma on public health, Link and Phelan (2006) conceptualize stigma as the result of an interconnected five-component process through which stigma is created and enforced. Within these components, i) people identify and label differences that are considered of consequence, ii) the labelled person is stereotyped with undesirable characteristics, iii) the group that applies the labelling differentiates the “them” (the stigmatized group) from the “us”, iv) the stigmatized people experience loss of status and discrimination, and v) there is an exercise of power that means those being stigmatized lack the economic, political, social, or cultural power to reverse the stigma. This conceptualization enables and facilitates sociological analyses of macro-level or structural explanations for and effects of stigma that often concentrate on understanding the role stigma plays in the creation and (re)production of social and structural inequalities and injustices (Tyler and Slater 2018) to inform contemporary public health understandings and potential responses.

In developing a useful conceptual tool for deconstructing, in-depth, the mechanisms of stigma as an instrument of power, Link and Phelan (2014, 24) propose the concept of stigma power to describe stigma as a resource for those with a vested interest in keeping a group of (Other) people “down, in or away.” In this sense, processes of stigma work to exploit, manage, control, or exclude those who are stigmatized, often in indirect, subtle, or hidden ways as part of “taken-for-granted cultural circumstances” (Link and Phelan 2014, 24). Link and Phelan draw on Bourdieu’s (1987) understanding of stigma as a form of symbolic power. For Bourdieu (1987), power is exercised through the creation of cultural distinctions of value and worth. Stigma “represents a statement about value and worth made by the stigmatisers about those they stigmatise” (Link and Phelan 2014, 25), which can be internalized by the stigmatized, and maintained through the boundary work of stigmatizers to control the social field, including the distribution of resources (Butler 2015). These statements, and the distinctions created between groups, are embedded surreptitiously within cultural systems of meaning that shape social structures.

Relatedly, sociologists, often drawing on the work of Foucault, focus on the structural effects and consequences of stigma(tization) and discrimination within and for societies. Following Foucault’s (1978) theorization that power is exercised through social norms, stigma can be understood as a tactic or strategy of power for maintaining and enforcing social norms and the established social order (Carrasco et al. 2017; Rose 1999). Parker and Aggleton (2003) used such perspectives to construct a conceptual framework through which to understand the effects, from micro to macro, of stigma and inform resistance approaches. This work argues that stigmatization and discrimination are social processes that “function, quite literally, at the intersection between culture, power and difference” to (re)produce relations of power and social inequalities. Building on this scholarship, Tyler and Slater (2018) introduce an important collection of works that, combined, reconceptualize the sociology of stigma.

In their analysis of stigma’s role and function in contemporary societies, Tyler and Slater (2018) present it as a form of classificatory power that, through the process of differentiation, reproduces and legitimizes various forms of social and structural inequalities and injustices. Stigma, as an organizing concept, provides a way of identifying, categorizing, and understanding different forms of discriminatory practices and attitudes that can be activated at different levels—through personal stories and experiences, social interactions, and at the structural level, through government and policy and media instruments (Tyler and Slater 2018). In this sense, “stigmatisation is [neither] a static nor a natural phenomenon, but rather a consequential and injurious form of action through collective representation fastened on people and on places” (Tyler and Slater 2018, 740). In this paper, we are particularly interested in the latter and how place stigma perpetuates enduring patterns of spatial injustice and health inequalities.

## Stigma and Place

Places carry both material resources and symbolic meanings (Agnew 1987; Gieryn 2000; Keene and Padilla 2014; Malatzky, Cosgrave, and Gillespie 2020a; Massey 1995). Thus, social inequalities can be represented geographically (Keene and Padilla 2014). There is a rich body of work, albeit exclusively from urban-focused perspectives, investigating the relationship between stigma and place (Tyler and Slater 2018). In a formative strand of this work, Wacquant (2007) argues that territorial stigmatization is a critical marker of “advanced marginality,” defined as a new(er) form of (urban) poverty caused by the segregation that contemporary global economic and neoliberal political systems encourage and often facilitate within local communities. Here, Wacquant extends Goffman’s earlier conceptualization of tribal stigma, that is, stigma associated with specific social constructs or meaning systems such as race, religion, or nation, and adapts Bourdieu's (1991) theory of symbolic power to examine how often “bounded territories” become perceived “by both outsiders and insiders as social purgatories, leprous Badlands,” “penalized spaces” (Wacquant 2007, 67), “zones reserved for [urban] outcasts” (Wacquant 2007, 68), and are subject to discourses of vilification, which are often internalized. In this process, Wacquant (2007, 67) describes how “*a blemish of place* [emphasis original] is . . . superimposed on the already existing stigmata traditionally associated with poverty and ethnic origin or postcolonial immigrant status.” Once this occurs, those living in stigmatized places can, in a more ready manner than others, be subject to special measures by authorities that are otherwise outside of accepted norms and practices and often, with some intent, reinforce marginalization and invisibility. For Wacquant et al. (2014), the development of territorial stigmatization necessarily involves symbolic societal structures that participate in the production of inequality and marginality. Relatedly, Halliday et al. (2021) emphasize “what leads an area to become stigmatised is closely aligned with its history as well as its socio-economic and political context.”

Working from a different disciplinary location, Halliday et al. (2020) have defined the concept of spatial stigma as the ways in which particular localities, and those who live in those localities, are negatively (re)presented and stereotyped in public, official, and political discourses. This includes mass social and news media, which are central channels through which many people come to perceive and understand the social world (Robertson 1987; Schiffman et al. 2005). Discrimination and prejudice spread when rural places and people are framed negatively in these forms of media. In this sense, dominant media can be understood as social structures that produce and perpetuate stigma (Corrigan et al. 2005). This is well understood in relation to many health and social issues, such as HIV (Taylor 2001), mental illness (Ross et al. 2021), and obesity (Couch et al. 2015).

Places can be stigmatized by their physical features and facilities (or lack thereof) and by perceptions of the “types” of people who live there. In addition to towns, wards, and estates as examples of the kinds of localities in which spatial stigma can be examined, the potential applicability of spatial stigma has been explored in relation to large-scale urban “redevelopment” or regentrification projects in which residents are often portrayed as somehow deficient so that their eventual displacement can be viewed as justified (Halliday et al. 2020; Kallin and Slater 2014; Paton, McCall, and Mooney 2017). It has also been argued that contemporary debates about policies of austerity are likely to exacerbate instances of spatial stigma (Halliday et al. 2020; Pearce 2012).

Halliday et al. (2020) have also noted the, to date, more extensive use of area reputation, especially in the United Kingdom, as a concept through which to examine the ways that place affects health in the field of public health. The reputation of an area can be either positive or negative and thus have protective or damaging effects on health. While acknowledging the utility of this concept, Halliday et al. (2020) argue that spatial stigma is a key structural driver of health inequalities and must be explicitly and substantially addressed within the field of public health. They suggest the principal reasons spatial stigma remains under-examined in public health include the limited focus on symbolic place meanings and how these inform health within the field and a “persisting failure to give weight to residents’ experiential knowledge of health inequalities in public health decision-making” (Halliday et al. 2020, 40). Further, Halliday (2020) and others (Smith and Anderson 2018; Thompson, Pearce, and Barnett 2007) emphasize that how public health entities communicate about health inequalities contributes to the perpetuation of spatial stigmas. This is largely done by focusing on individual behavioural explanations rather than socio-structural conditions that constrain people’s options. In this sense, Whittaker et al. (2020) highlight how policies and “initiatives” can reproduce and exacerbate place-based stigmas.

For Keene and Padilla (2014), the concept of spatial stigma provides a means to dissect how social meanings and cultural logics manifest in places and how these manifestations are intrinsically influenced by broader political, structural, and cultural systems. In synthesizing a broad range of literature, these researchers propose three primary pathways through which spatial stigma and the health of those living in stigmatized places connect. Firstly, spatial stigma influences people’s access to a wide range of resources needed to achieve, sustain, and promote health (access to resources). Spatial stigma affects people’s access to services; it can lead those who provide services to make stereotypical assumptions about people living in stigmatized places, limit educational and employment opportunities, and impact investment and disinvestment in communities. Secondly, spatial stigma is a source of chronic stress, including psychosocial stress, which severely affects physical, mental, and emotional health (stress and coping). People living in or associated with stigmatized places report poorer levels of satisfaction with health and life in general. They are more likely to be diagnosed with a mental health condition. Thirdly, the extent to which negative perceptions of place can become internalized undermines well-being. The labour involved in managing the experience of spatial stigma has important consequences for identity formation, people’s social relationships, social connections, and sensations of belonging (identity formation and management). In shining a spotlight on the bodies of evidence around how spatial stigma effects contribute to the creation and (re)production of health inequalities, Keene and Padilla (2014) emphasize the importance of critically interrogating dominant discourses about marginalized places in popular media and academia. This is part of raising social consciousness about how spatial stigma “circulates, whom it benefits, and how it functions— through the very act of its articulation—to reinforce social inequalities in vilified places” (Keene and Padilla 2014, 401).

With a similar objective, Bambra (2022) makes a strong case for place to be treated as an aspect of intersectionality—as an aspect of social identity—in intersectional analyses of health inequalities. This is an important argument to consider when research on spatial and territorial stigma has been almost entirely focused on urban place contexts. Rural places are rarely considered, yet in many countries, including Australia, the stigmatization of rural places is common. In this way, rurality, as a kind of place, can be a marker of social identity that marginalizes inhabitants of rural places. We seek to develop an understanding of the power of rural place stigma in order to address the implications for rural people.

## The Stigmatization of Rural Places

The divisive effects of contemporary global economic and neoliberal political systems responsible for the advanced marginality that Wacquant (2007) described in highly urbanized settings have also profoundly affected rural societies (Malatzky and Smith 2022). While place is relational, situated within complex networks of social meanings, social relations, and power struggles (Cummins et al. 2007), “rural” and “urban” places are still commonly presented as dichotomous in many policy contexts and media descriptions (Jansson 2012). These portrayals of place enable rural places to be persistently positioned on the periphery of public and political debates. In addition, the place-determinant effects of global forces on rural places and the different needs of rural people and communities are largely ignored or approached paternalistically by national policymakers (Malatzky and Smith 2022; Pini, Rodriguez Castro, and Mayes 2021). A common technique involved in the process of relegating the rural from broader political attention and controlling how rural matters are considered is the ongoing construction and treatment of rural places as homogeneous. In their systematic review of how rurality is defined and used in empirical, quantitative research, Nelson et al. (2021) point to how the process of implementing rural policies is frequently driven by quantitative measures that implicitly assume that rural places are homogenous and experience no variations in conditions. This assumption erases the heterogeneity of rural places (Dalsgaard Pedersen and Gram 2018; Rye 2006).

The nature of rural places is dependent on a range of social processes and material concerns, including the location of a rural place relative to larger metropolitan centres; the local economic structure and activities, including the types of local industries that dominate in a particular rural locale—for example, whether it is a farming, manufacturing, service sector, or tourism town; the nature of the physical environment, inclusive of topography, climate, and other natural conditions or features; human resources or population profile; the type and quality of in-place infrastructure, including those that connect places; and the degree to which a rural place is embedded within broader networks (Isserman, Feser, and Warren 2009; Li, Westlund, and Liu 2019; Marsden 1999; Meijers and Van der Wouw 2019). Thus, the kinds of social and economic opportunities and resources available to residents-in-place vary between different rural places. For example, some rural places may have fewer opportunities for stable employment than others, and there can be significant variation in the kinds of, and diversity within, local economic and social institutions (Bernard 2019).

Relatedly, some rural places succeed in being categorized as “cosmopolitan country,” where rural idyll exists with urban(e) features and benefits (Gorman-Murray, Waitt, and Gibson 2012; Malatzky et al. 2020a, 2020b) and support city-like consumption through restaurants, cafes, shopping, and beauty and spa treatments. In studying rural and regional youth in Australia, Farrugia (2020, 238) notes that “the cultural politics of class interacts with the social and economic history of particular localities to produce grammars of place that *either stigmatise or valorise* [emphasis added] local places and young classed identities.” In this sense, not *all* rural places are stigmatized. For example, some rural places are associated with prestige, and occupancy signifies wealth and privilege. In these cases, a rural place can be used by those with the financial resourcing to do so as a tool in the creation of a socially desirable identity, to “act as an important marker of identity and sense of identification” (Hopkins 2010, 11). However, many rural places are stigmatized in various interconnecting ways that have yet to be systematically examined in public or rural health research.

Many dominant stereotypes and tropes used to describe rural places and people in popular media, political, and, sometimes, academic discourses are stigmatizing. Rural places are frequently constructed as inferior and lacking compared to metropolitan or urban places—rural places are boring, dull, disadvantaged, and in decline (Isserman, Feser, and Warren 2009; Malatzky and Bourke 2016). Rural places are also commonly portrayed as sites of poverty, where people with low levels of educational attainment reside, where there are fewer and poorer quality services, limited infrastructure, a lack of “culture” and opportunity, and high degrees of conservatism and are considered a “burden” to the taxpayer (Malatzky and Bourke 2016; Skogen and Krange 2003). Rural people are frequently labelled as backwards, uneducated, and parochial (Malatzky and Bourke 2016; Sandberg 2013). The long-running (1962–1971) American sitcom, *The Beverly Hillbillies*, where “a nouveau riche hillbilly family moves to Beverly Hills and shakes up the privileged society with their hayseed ways” (*The Beverly Hillbillies* 2022), was an early example of entertainment media juxtaposing the tensions and assumptions about city and rural and positioning rural people as unsophisticated and backwards.

Similarly, rural Australia has been romanticized in many Australian movies, such as *The Man from Snowy River*, and television series like *The Flying Doctors*. However, there is often an undercurrent of the rural as a little wild, risky, untamed, uncivilized, and uncouth. The juxtaposition of a rural person meeting a city person is a tried-and-true formula for casting rural people as either country bumpkins, rednecks, unfashionable, naive or a little “slow.” These assumptions are built into the English language. Both *urban* and *urbane* derive from the Latin word for city, *urbs.* Urban means of, relating to, characteristic of, or constituting a city (Merriam-Webster 2022a), and urbane means notably polite or polished in manner (Merriam-Webster 2022b).

Social media also contributes to some of this stigmatization. *Sht Towns of Australia*, a “comedy” Facebook page, is described as “The foremost authority on shit towns in Australia,” with around 409,000 page likes and 582,000 followers in October 2022 (*Sht Towns of Australia* 2022). There is also an accompanying book of the same title (Furphy and Rissole 2019). While this page and book do not exclusively focus on rural locations, the majority of locations listed are rural or regional. The page reviews Australian towns, “publishing a barrage of their hate mail and compiling weekly lists of the worst news stories from across the nation” (Moussalli 2019). The page is positioned as comedic, but the posts and critiques of locations raise the ire of many people living in those locations while also feeding into more stigma as others share comments and views on why a location is so terrible. The high profile of this Facebook page also means that the annual announcement of the “winning” town often receives mainstream media attention (Brown 2020). In this way, media as a techno-social system provides information that is “produced, distributed and consumed with the help of technologies in a dynamic and reflexive process that connects technological structures and human agency” (Fuchs 2017, 40).

The kinds of intersections with race and class stigma that others have observed in cases of territorial or spatial stigma in urban settings, although less explicit, are still manifest within popular representations of rurality and rural people (Halliday et al. 2020; Keene and Padilla 2014; Wacquant 2007; Wacquant, Slater, and Pereira 2014). Morris (2012) observed these intersections in an examination of how young people from low-income families manage race and class-based inequalities in rural Ohio. In this study, some small rural towns were stereotyped as the homes of “rednecks,” “hillbillies,” or “white trash.” Isserman et al.’ (2009) analysis of why some rural places prosper and others do not alludes to the racialized dimensions of these interactions in their finding that rural places with fewer Hispanic, African American, or Indian American residents were more “prosperous”—when in fact, these places had better access to resources needed for prosperity—than those with greater racial diversity. In the Australian context, rural places where perceivably large numbers of First Nation Australians reside are positioned in similar ways (D’Abbs 2012; Fforde et al. 2013). These stigmatizing representations of rurality are experienced by those living in rural places. For example, political journalist and writer Gabrielle Chan, who also left urban Australia to live rurally, writes about her rural experiences and reflections in the book (2018) *Rusted Off*, including how rural people experience “urban splaining” where city people talk down to them and that there is a persistent geographical judgement placed on rural people.

These social and symbolic meanings attached to many rural places and people illustrate the interconnected five-component process through which stigma is created and maintained described by Link and Phelan (2006). Rural places and people are identified and labelled as different to the norm, stereotyped as undesirable, and juxtaposed against dominant constructions of urban places as the epicentres of sophistication, class, and progress (Malatzky and Bourke 2016). For example, when discussing her experiences of managing a career from a rural location, journalist Kirsten Diprose (2019) explained:

Many people living in the country feel they have to justify their careers, whether it’s in the media industry, health, education or business. Some people think if you’re not working in the metropolitan centres, then you must not be good enough at what you do. You never cracked the “big time” or you were too afraid to try.

In the same news article, another rural woman, Dr Kristy Hess, explained how rural and regional people internalize this “inferiority complex,” and that “regional areas are perceived as ‘lesser,’” which feeds into the acceptability of the idea “that regional people move to the city for ‘opportunity’” (Diprose 2019).

Other news stories have highlighted that rural young people can experience a sense of failure if they choose not to “move to the bright city lights, to the land of multi-lane highways, merging and public transport,” or if they move away and then return, they are faced with comments such as “Why would you choose to come back?” (Butterworth 2019), or “What are you doing with your life?,” with the underlying assumption that returning to a rural or regional area means your life is “going nowhere” (Rääbus 2019). These types of experiences appear not to be limited to the Australian context. Rainesford Stauffer (2021), a freelance writer in Kentucky in the United States, also writes that after leaving, her returning “felt like ‘going back’ got framed as quitting,” especially when moving away is seen as “a rite of passage,” particularly when moving to the “places exalted as the centers of ultimate young-adult experience—big cities and college towns.” Others explain how these assumptions that rural life and opportunities are lesser are reinforced through educational experiences. “The message at school was: ‘If I wanted to have an interesting life or be successful, I needed to move away and go to the city’” (Rääbus 2019).

The implications of stigmatizing rural places also evidence the three primary pathways, described by Keene and Padilla (2014), through which spatial stigma and the health of those living in stigmatized places connect. For example, many rural places in Australia and other high-income economy countries experience significant workforce shortages in areas such as education and health (Cosgrave 2020; Downes and Roberts 2018), which affect people’s access to health services and quality education—key resources required to foster and sustain good health. The ongoing ramifications for the health and well-being of rural people from the maldistribution of the health and social care workforce are well documented in contemporary literature. It is a core contributor to persistent health disparities between those who live in rural as opposed to metropolitan places (Australian Institute of Health and Welfare 2022; Simpson and McDonald 2017; World Health Organization 2021).

The dominance of negative constructions and understandings of rural places is also likely to contribute to the challenges of attracting, recruiting, and retaining professionals in rural communities. However, there has been little focus on perceptions of rurality and if and how this might impact workforce decisions. Little attention has been given to how government policy reinforces deficit views of rural. For example, how systems for classifying types of places like the Modified Monash Model in the Australian context imply systemic deficits in rural communities or how policies across different levels of government, such as state-level health infrastructure and national-level rural medical schools in Australia, may be influenced by rural place stigma and perpetuate *parens patriae* style approaches to governing the rural.

Further, how young people, in particular, talk about rural places exemplifies the internalization of place stigma and the subsequent effects on well-being, which to date has mostly been examined in urban place contexts (Halliday et al. 2020). Phoebe Nagorcka-Smith, a young Australian from Portland, Victoria, provides a clear example of how young people can internalize the stigma of rurality.

The stigma for me was, if I’m thinking as a young person that I want to do something that really makes a difference, am I able to do that in a rural area? Does my work matter? … [It was based on] a bit of an assumption that whatever I could do and whatever difference I made in my community, it wouldn’t be worth as much because it was a rural community compared to a metro one. (Rääbus 2019)

Along with the issues raised by rural and regional young people, rural health professionals can also experience an implicit stigma from their urban colleagues. This is exemplified when they are asked why they left the city and when they will be returning, informed by assumptions that “nobody would voluntarily move to a country town for professional work, especially if they have no family or social ties to the area” (Baker and Hess 2019), and through the common way that rural doctors are considered to be second-rate failures, and “that no doctor would honestly choose to work in the bush” (Plevey 2022). These examples of direct personal experience are consistent with research suggesting that, despite rural medicine being broader in both scope and complexity, it is often considered low status (Malatzky and Bourke 2016). Yet rural practitioners highlight the richness and positive experiences of their rural health work (Couch et al. 2020). This suggests that the perspectives, experiences, and knowledge of those residing in rural places can radically differ from those articulated within dominant discourses. We now turn to rural people’s responses to and the possibilities for dismantling rural place stigma.

## Responses to Rural Place Stigma

Halliday et al. (2020) describe how residents of stigmatized places can and do develop strategies for challenging and resisting stigmatizing portrayals of their communities. These strategies centre on providing and promoting alternative understandings of and narratives about the place within the broader domain that are based on residents’ local knowledge and experiences. Nayak (2019, 944) argues that residents use these to “‘speak back’ to the dominant regimes of representation that seek to define them.” Community initiated and led campaigns and partnerships, often related to local art and festivities, are sites through which these alternative narratives are often taken up. In the context of the stigmatization of rural places, the *Australian Silo Art Trail* initiative (2021) and the Regional Australia Institute’s *Move to More* campaign (2022) are examples of utilizing and promoting alternative understandings and attributes of rural places, derived from within rural places, in the broader public and media domain. The use of these kinds of alternative narratives by rural residents as a form of resistance to the stigmatization of rural places is an attempt to “re-script” rural places in the broader social imaginary (Nayak 2019), and by doing so, address the implications of the stigmatization of rural places for health and social inequalities at the community level (Halliday et al. 2020).

Importantly, however, rural place stigma is produced by deep structural level inequalities that are (re)produced through multiple institutional and policy mechanisms (Halliday et al. 2020). As Whittaker et al. (2020, 3) highlight, “spatial stigma is maintained by an *outside* gaze looking in” rather than the actions of local actors. Thus, there is a limit to how effective community-level responses can be in remedying the stigmatization of rural places. This is especially important to consider given the subtle mechanisms of stigma power (Link and Phelan 2014) that can lead to some community-focused initiatives surreptitiously feeding back into dominant relations of power and reproducing spatial stigma (Whittaker, Tran, and Keene 2020). For this reason, Halliday et al. (2020, 41) emphasize that the residents of stigmatized places are “not responsible for dismantling spatial stigma.” Rather, it is the responsibility of those in influential positions to inform political and institutional decision-making to prioritize the perspectives of these residents. For example, to address persistent patterns of spatial injustices and health inequalities between rural and metropolitan places in countries like Australia, policymakers need to critically consider and engage with how policy instruments may inadvertently or otherwise reinforce rural stigmas. Part of this process would involve a deliberate shift away from paternalistic approaches to rural development that assume those living in urban centres know what is best for rural communities towards models of governance that ascribe greater political autonomy and decision-making directly to rural residents. Ultimately, the social institutions involved in stigmatizing rural places—those with a vested interest in the stigmatization of rural places—must be held accountable for the (re)production of spatial and health inequalities experienced by rural residents.

## Conclusion

Rural place stigma has serious consequences for rural communities. It plays a key role in maintaining and perpetuating the kinds of spatial injustices and health inequalities experienced by those living in rural places. Consequently, the issue of rural place stigma and its relationship to the (re)production of power relations that marginalize and disadvantage the rural in public and political debates should be attended to, especially within public and rural health research and praxis. Public and rural health are central fields of inquiry tasked with addressing the socio-structural drivers of health inequalities largely inflicted on those who experience marginalization and exclusion within broader society, including those living in rural places. Rural place stigma has received little direct attention from within these fields. In this paper, we have outlined a case for the importance and relevance of stigma for the experiences of rural people and conditions within rural places. We have conceptualized stigma as a device of power embedded within and influencing social and political structures, norms, and processes. In presenting this case, we hope to open discussion and encourage and stimulate reflection on who and what contribute to rural place stigma. This will build energy for developing new bodies of critical public and rural health research that engages with the mechanisms and consequences of rural place stigma and provide ways of dismantling this at the systemic, structural level.
